# Cardiometabolic profile and leukocyte telomere length in a Black South African population

**DOI:** 10.1038/s41598-022-07328-8

**Published:** 2022-02-28

**Authors:** Ndonwi Elvis Ngwa, Tandi E. Matsha, Carl Lombard, Naomi Levitt, Eugene Sobngwi, Andre-Pascal Kengne, Nasheeta Peer

**Affiliations:** 1 Laboratory for Molecular Medicine and Metabolism, Biotechnology Center, University of Yaoundé 1, Yaoundé, Cameroon; 2 South African Medical Research Council/Cape Peninsula University of Technology Cardio-Metabolic Health Research Unit, Department of Biomedical Sciences, Faculty of Health and Wellness Sciences, Cape Peninsula University of Technology, Cape Town, South Africa; 3 Biostatistics Unit, South African Medical Research Council, Cape Town, South Africa; 4 Chronic Disease Initiative for Africa, Department of Medicine, UCT, Cape Town, South Africa; 5 Non-Communicable Diseases Research Unit, South African Medical Research Council, Durban, Cape Town, South Africa; 6 Department of Medicine, University of Cape Town, Cape Town, South Africa; 7 Division of Epidemiology and Biostatistics, Department of Global Health, University of Stellenbosch, Cape Town, South Africa

**Keywords:** Biomarkers, Cardiology, Diseases, Risk factors

## Abstract

Several studies have reported a possible association between leucocyte telomere length (LTL) and cardio-metabolic diseases (CMDs). However, studies investigating such association are lacking in South Africa despite having a very high prevalence of CMDs. We investigated the association between LTL and CMD risk profile in a black South African population. This was a cross-sectional study with participants > 21 years of age and residing in five townships in Cape Town. CMD markers were compared between men and women and across quartiles of LTL. Linear and logistic regressions relate increasing quartile and Log_10_LTL with CMD risk profile, with appropriate adjustment. Among 676-participants, diabetes, obesity and hypertension prevalence were 11.5%, 23.1% and 47.5%. Waist-circumference, hip-circumference and highly sensitive c-reactive protein values were significantly higher in women (all *p* < 0.001), while HDL-C (*p* = 0.023), creatinine (*p* = 0.005) and gamma glutamyl transferase (*p* < 0.001) values were higher in men. In age, sex and BMI adjusted linear regression model, Log_10_ of LTL was associated with low HDL-C (beta = 0.221; *p* = 0.041) while logistic regression showed a significant association between Log_10_LTL and prevalent dyslipidaemia characterised by high LDL-C. In this population, the relationship between LTL and CMD is weak given its association with only HDL-C and LDL-C.

## Introduction

Telomeres are specialized chromosomal DNA–protein structures that make up the terminal regions of chromosomes and are formed of a highly conserved hexameric (TTAGGG) tandem repeat DNA sequence. Their primary function is to protect and stabilize the genetic material carried by chromosomes in several ways. They shield the chromosomal termini from recognition by the DNA damage response system of the cell, cap chromosome ends thereby preventing them from being degraded or fused together. The length of the telomere is longest at birth and begins to decline within the first 6 weeks of life. In addition to chronological age, there is a wide range of genetic and environmental factors modulating telomere length (TL). Gender^1^, ethnicity^2^ and level of physical activity^3^ have been shown to modulate TL. Moreover, short TL has been shown to be associated with type 2 diabetes in Caucasian, South Asian and Afro-Caribbean^4^, Insulin Dependent Diabetes Mellitus (IDDM) in white American men^5^**,** gestational diabetes mellitus in Chinese population^6^, coronary artery disease in Chinese men^7^, vascular dementia in German population^8^, cardiovascular disease^9,10^ and hypertension^11^ in the Adult United State population. This large body of evidence therefore suggests that there is a possible relationship between TL and cardiometabolic diseases (CMDs).

South Africa is facing a quadruple burden of diseases including infectious diseases such as HIV and tuberculosis; maternal and child conditions; non-communicable diseases (NCDs); and violence, injuries and trauma^12^. CMDs, the leading NCDs, are among the top ten causes of death in South Africa^12^. Accordingly, identification of biomarkers of CMDs is essential for early diagnosis and appropriate management of these conditions especially in black South Africans marked by higher prevalence of CMDs. Given that CMDs have been shown to be associated with leucocyte telomere length (LTL) in European, American and Asian populations, we therefore undertook this study to investigate the association between LTL and cardio-metabolic profile in a black South African population.

## Results

Out of the 1116 participants examined in this study, DNA extracted from stored samples for analysis were available for 676 participants. This number resulted from samples with LTL values which could only be quantified on good quality DNA sample (absorbance 260 nm/280 nm ratio between 1.7 and 2.0). The prevalence of the cardiometabolic disorders was 47.5% for obesity, 23.1% for hypertension and 11.5% for type 2 diabetes (Table 1). Weight, BMI, heart rate, WC, HC and hs-CRP (all *p* < 0.001) median values were higher in women than men while height (*p* < 0.001), HDL-C (*p* = 0.023), creatinine (*p* = 0.005) and GGT (*p* < 0.001) values were significantly higher in men than women (Table 1). Moreover, more women than men were obese by all parameters measured [BMI (*p* < 0.001), WC (*p* < 0.001), WHR (*p* = 0.012) and WHtR (*p* < 0.001)] and had dyslipidaemia [high triglycerides (*p* = 0.003), high LDL-C values (*p* = 0.009) and high non-HDL-C values (*p* = 0.03)].

**Table 1:** Cardiometabolic characteristics of the study population.

Variable	Total N = 676	Men 231	Women 445	*p* Value
**Median values (25th–75th percentiles)**				
Age (years)	42.0 (32.0–53.0)	41.9 (31.0–54.0)	43.0 (32.0–52.0)	0.467
Weight (kg)	77.1 (63.6–92.0)	65.2 (58.3–76.2)	83.8 (69.6–99.4)	< 0.001*
Height (cm)	162.5 (157.0–168.5)	170.0 (165.5–175.0)	159.0 (155.0–163.5)	< 0.001*
Body mass index (kg/m^2^)	29.0 (23.1–35.5)	22.2 (19.9–25.9)	32.9 (27.5–38.5)	< 0.001*
Waist circumference (cm)	92.5 (80.5–105.0)	80.9 (75.5–92.2)	97.4 (88.0–108.0)	< 0.001*
Hip circumference (cm)	107.0 (95.1–121.0)	93.5 (88.45–100.9)	115.7 (104.9–125.5)	< 0.001*
Waist-to-hip ratio (WHR)	0.86 (0.81–0.90)	0.88 (0.83–0.94)	0.85 (0.80–0.89)	0.469
Waist-to-height ratio (WHtR)	0.57 (0.48–0.65)	0.48 (0.44–0.54)	0.61 (0.55–0.69)	0.384
SBP (mmHg)	121.0 (108.5–134.5)	126.0 (114.2–139.2)	116.5 (106.5–130.5)	0.304
DBP (mmHg)	79.5 (72.0–89.0)	79.5 (72.5–89.5)	79.0 (71.5–88.5)	0.283
Heart rate (beats/ min)	68.5 (61.0–78.0)	63.0 (56.0–71.5)	71.75 (64.5–80.0)	< 0.001*
Total cholesterol (mmol/L)	4.27 (3.61–5.03)	4.28 (3.54–5.00)	4.28 (3.65–5.08)	0.563
HDL-C (mmol/L)	1.05 (0.89–1.32)	1.12 (0.93–1.41)	1.04 (0.87–1.26)	0.023*
Triglycerides (mmol/L)	0.95 (0.70–1.29)	0.98 (0.71–1.39)	0.95 (0.70–1.24)	0.180
LDL-C (mmol/L)	2.94 (2.36–3.61)	2.79 (2.15–3.41)	3.02 (2.44–3.69)	0.179
Non HDL-C (mmol/L)	3.16 (2.56–3.87)	2.99 (2.39–3.74)	3.24 (2.64–3.91)	0.525
Fasting glucose (mmol/L)	4.8 (4.4–5.3)	4.9 (4.4–5.3)	4.8 (4.3–5.3)	0.579
2-h glucose (mmol/L)	5.8 (4.8–7.5)	5.9 (4.65–7.5)	5.8 (4.9–7.5)	0.607
Urea (mmol/L)	3.5 (2.7–4.3)	3.5 (2.7–4.5)	3.5 (2.8–4.2)	0.406
Creatinine (μmol/L)	57.0 (46.0–71.0)	64.0 (51.0–79.0)	54.0 (43.0–64.0)	0.005*
hs-CRP (mg/L)	3.36 (1.22–7.96)	1.98 (0.74–5.38)	4.29 (1.54–10.08)	< 0.001*
GGT (U/L)	25 (16–43)	32 (21–55)	22 (14–37)	0.012*
Telomere length (kb)	52.3 (37.3–71.6)	52.7 (36.2–73.4)	51.7 (38.0–70.9)	0.516
**Prevalence, n (%)**				
Adiposity				
BMI ≥ 30 kg/m^2^	321 (47.5)	28 (12.1)	293 (65.8)	< 0.001*
Waist circumference (WC): men > 94 cm, women > 80 cm	435 (64.4)	46 (19.9)	389 (87.42)	< 0.001*
Waist to hip ratio (WHR) ≥ 0.9 in men and ≥ 0.85 in women	285 (42.2)	80 (34.6)	205 (46.1)	0.012*
Waist to height ratio (WHtR) > 0.5	471 (69.7)	80 (34.6)	391(87.9)	< 0.001*
Hypertension	156 (23.1)	55 (23.8)	101 (22.7)	0.75
Diabetes	78 (11.5)	25 (10.8)	53 (11.9)	0.41
**Dyslipidaemia**				
TC > 5.0 mmol/L	163 (24.1)	53 (22.9)	110 (24.7)	0.391
HDL-C < 1.2 mmol/L	449 (66.4)	137 (59.3)	312 (70.1)	*0.006
Triglyceride > 1.5 mmol/L	99 (14.6)	48 (20.8)	51 (11.5)	*0.003
LDL-C > 3.0 mmol/L	290 (42.9)	85 (36.8)	205 (46.1)	*0.009
Non HDL-C > 3.37 mmol/L	266 (39.4)	79 (34.5)	187 (42.0)	*0.03

Legend: *SBP* systolic blood pressure, *DBP* diastolic blood pressure, *WC* waist circumference, *WHR* waist to hip ratio, *WHtR* waist to height ratio, *HDL-C* high-density lipoprotein cholesterol, *LDL-C* low-density lipoprotein cholesterol, *GGT* gamma glutamyl transferase, *hs-CRP* highly sensitive c-reactive protein.

* means there is significant difference in the variable comparing men with women.

**Figure 1:**
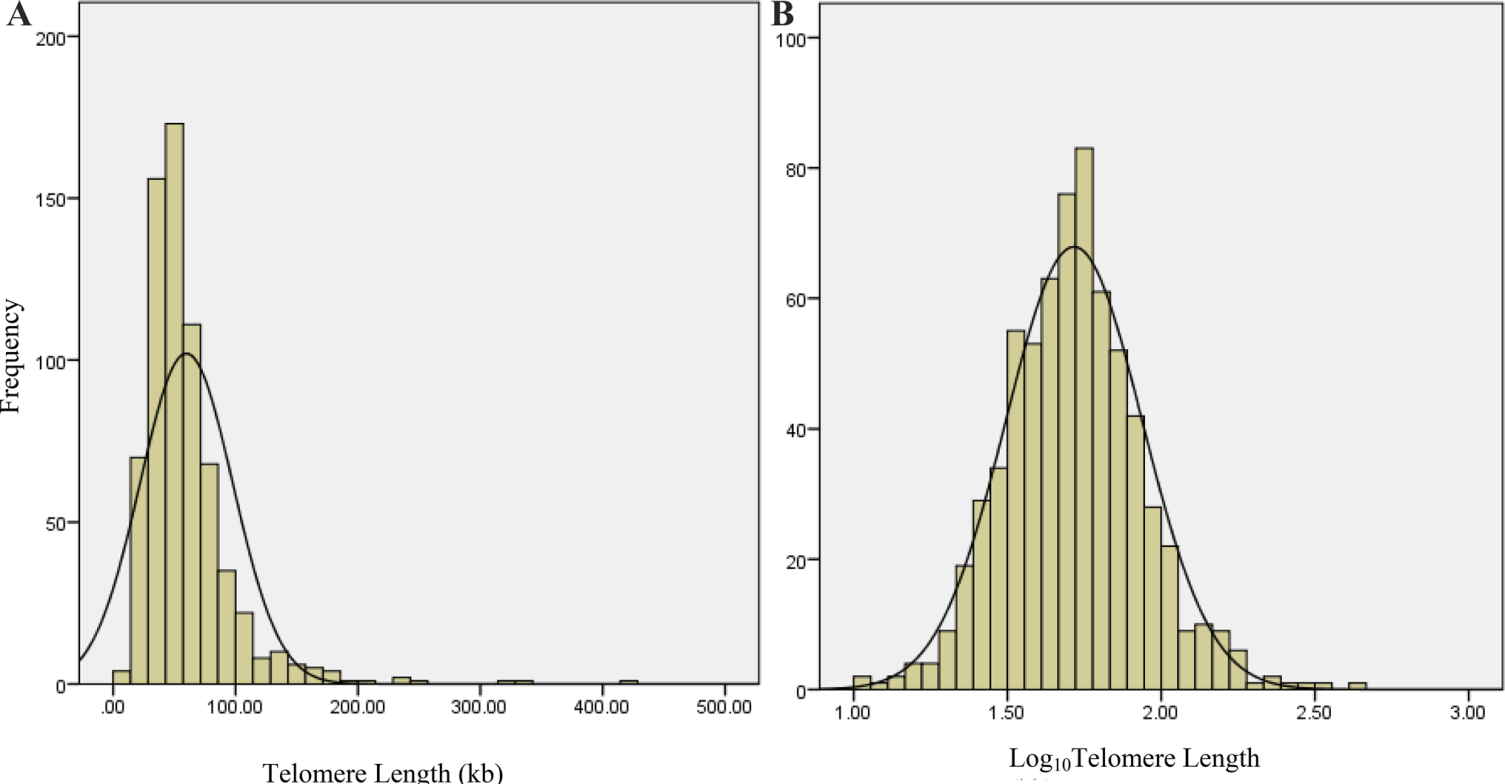
(**A**) Distribution of telomere length in the general population, (**B**) Distribution of Log_10_ telomere length in the general population.

The distribution of TL was skewed to the right (skewness = 3.45), indicating that TL values were not normally distributed (Fig. 1A). In order to have a normal distribution of TL, it was Log_10_ transformed (Fig. 1B).

**Figure 2:**
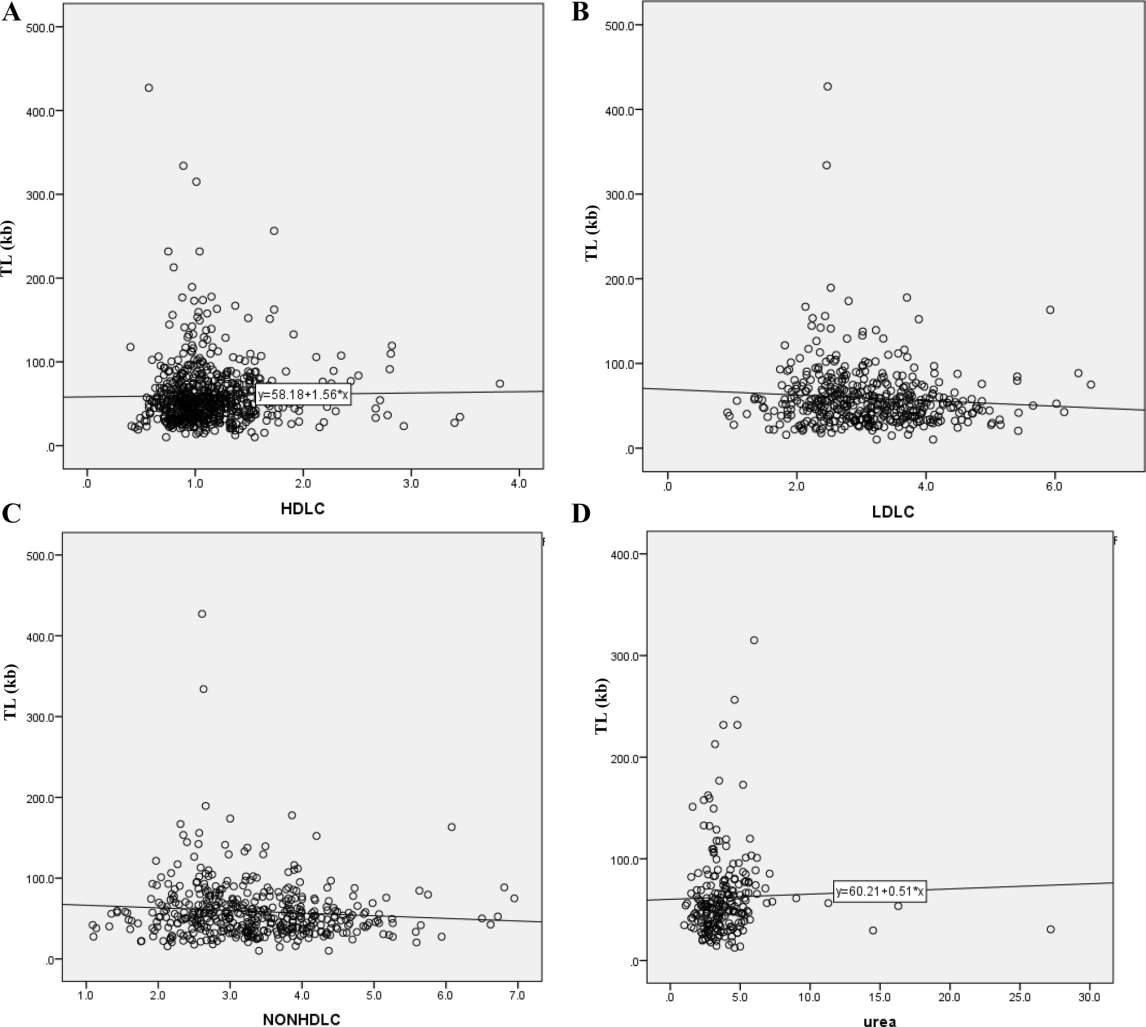
(**A**) Correlation between LTL and HDL-C in the general population. (**B**) Correlation between LTL and HDL-C in Females. (**C**) Correlation between LTL and non-HDL cholesterol in female. (**D**) Correlation between LTL and urea in male.

Correlation analysis showed a significant positive association between LTL and HDL-C (r = 0.082, *p* = 0.034) (Fig. 2A). In women, LTL was negatively correlated with LDL-C (r =  − 0.1, *p* = 0.037) (Fig. 2B) and non-HDL-C (r =  − 0.101, *p* = 0.034) (Fig. 2C). In men, there was a significant positive correlation between LTL and Urea (r = 0.179, *p* = 0.007) (Fig. 2D).

The association between LTL and cardiometabolic profile was investigated by categorizing the LTL values into quartiles with the first being the lowest and the fourth being the highest (Table 2). Amongst the different cardio-metabolic parameters investigated, quartiles of LTL were shown to be positively and significantly associated with HDL-C (Table 3).

**Table 2:** Distribution of telomere length in quartiles.

	Quartile 1 (n = 170)	Quartile 2 (n = 171)	Quartile 3 (n = 169)	Quartile 4 (n = 170)
Mean SEM	28.53 ± 0.46	44.80 ± 0.33	59.87 ± 0.41	106.11 ± 3.71
Median	29.50	44.89	59.05	88.93
Range	27.06	15.03	18.04	356.57
Interquartile range	8.45	7.51	8.78	34.06

**Table 3:** Cardio-metabolic profile presented by telomere length quartiles.

Variable	Telomere length category				*p* Value for linear trend	Correlation coefficient (2-tailed significance)
	1st quartile Median (25–75)	2nd quartile Median (25–75)	3rd quartile Median (25–75)	4th quartile Median (25–75)		
n	169	169	169	169		
**Median values (25th–75th percentile)**						
Age (years)	43 (32–54)	41 (32–52)	40 (32–40)	44 (32–55)	0.509	0.016 (0.675)
Weight (kg)	77.1 (63.4–77.1)	76.2 (62.8–90.7)	79.3 (62.1–95.1)	74.8 (64.1–89.3)	0.848	− 0.003 (0.931)
Height (cm)	164.0 (158.0–171.5)	163.0 (157.4–166.5)	162.5 (156.8–168.1)	162.4 (156.5–169.5)	0.345	− 0.041 (0.286)
Body mass index (kg/m^2^)	28.6 (22.4–35.4)	29.3 (23.2–35.2)	28.9 (22.9–34.8)	28.7 (22.9–34.8)	0.888	0.007 (0.853)
Waist circumference (cm)	93.0 (79.9–106.1)	91.5 (80.7–103.0)	93.8 (80.5–107.4)	91.5 (80.0–103.4)	0.774	− 0.011 (0.782)
Hip circumference (cm)	107.5 (94.4–122.0)	109.0 (95.6–119.0)	106.9 (95.4–112.7)	105.0 (95.5–118.5)	0.758	− 0.017 (0.663)
Waist to hip ratio (WHR)	0.87 (0.82–0.91)	0.85 (0.80–0.89)	0.86 (0.81–0.91)	0.85 (0.81–0.91)	0.825	− 0.032 (0.409)
Waist to height ratio (WHtR)	0.57 (0.49–0.66)	0.56 (0.50–0.65)	0.57 (0.49–0.67)	0.57 (0.48–0.64)	0.939	− 0.004 (0.926)
SBP (mmHg)	119.3 (106.9–134.0)	122.5 (108.0–133.7)	121.0 (110.1–133.5)	122.5 (110.5–138.5)	0.140	0.055 (0.154)
DBP (mmHg)	77.5 (71.4–89.1)	80.3 (72.4–88.0)	79.5 (73.0–89.0)	79.0 (70.5–90.5)	0.417	0.037 (0.339)
Heart rate (beats/min)	69.5 (62.4–77.1)	67.5 (60.4–78.0)	70.3 (61.1–80.4)	68.5 (61.0–77.5)	0.909	− 0.003 (0.933)
Total cholesterol (mmol/L)	4.3 (3.7–4.9)	4.4 (3.5–5.3)	4.3 (3.7–5.0)	4.2 (3.6–5.1)	0.436	0.011 (0.772)
HDL-C (mmol/L)	1.02 (0.86–1.28)	1.04 (0.89–1.28)	1.09 (0.88–1.31)	1.07 (0.93–1.39)	0.041*	0.092 (0.017) *
Triglycerides (mmol/L)	0.95 (0.71–1.32)	0.98 (0.74–1.24)	0.94 (0.68–1.28)	0.98 (0.67–1.24)	0.991	− 0.003 (0.932)
LDL-C (mmol/L)	2.97 (2.37–3.55)	3.05 (2.35–3.75)	2.86 (2.35–3.58)	2.86 (2.37–3.57)	0.973	− 0.021 (0.586)
Non HDL-C (mmol/L)	3.23 (2.57–3.79)	3.24 (2.54–4.00)	3.07 (2.56–3.83)	3.03 (2.57–3.81)	0.902	− 0.026 (0.506)
Fasting glucose (mmol/L)	4.90 (4.40–5.40)	4.80 (4.48–5.20)	4.70 (4.30–5.30)	4.80 (4.30–5.50)	0.805	− 0.014 (0.719)
2-h glucose (mmol/L)	5.80 (4.78–7.43)	5.65 (4.78–7.13)	5.85 (4.73–7.50)	6.10 (4.90–7.90)	0.515	0.035 (0.372)
Urea (mmol/L)	3.4 (2.7–4.3)	3.5 (2.6–4.4)	3.45 (2.6–4.1)	3.6 (3.0–4.4)	0.447	0.061 (0.112)
Creatinine (μmol/L)	56.0 (44.0–71.3)	55.5 (45.0–69.0)	56.5 (45.0–67.8)	58.0 (49.0–76.0)	0.201	0.067 (0.085)
hs-CRP (mg/L)	3.89 (1.29–9.29)	3.05 (1.30–6.94)	3.49 (0.98–8.15)	2.75 (1.21–8.53)	0.140	− 0.030 (0.432)
GGT (U/L)	26 (15–43)	23.0 (16.0–37.0)	25.5 (16.0–47.5)	25.0 (15.0–47.0)	0.780	0.024 (0.532)
**Prevalence, %**						
Body mass index ≥ 30 kg/m^2^	82 (48.5)	78 (46.2)	81 (47.9)	80 (47.3)	0.976	− 0.004 (0.918)
Waist circumference (WC): men > 94 cm, women > 80 cm	106 (62.7)	117 (69.2)	107 (63.3)	105 (62.1)	0.499	0.013 (0.687)
Waist to hip ratio (WHR): men ≥ 0.9, women ≥ 0.85	77 (45.6)	66 (39.1)	77 (45.6)	65 (38.5)	0.385	0.029 (0.411)
Waist to height ratio (WHtR) > 0.5	118 (69.8)	121 (71.6)	119 (70.4)	113 (66.9)	0.671	0.020 (0.571)
Hypertension	40 (23.7)	34 (20.1)	39 (23.1)	43 (25.4)	0.706	− 0.021 (0.574)
Diabetes	17 (10.1)	15 (8.9)	23 (13.6)	23 (13.6)		
**Dyslipidaemia**						
Total cholesterol > 5.0 mmol/L	37 (21.9)	46 (27.2)	39 (23.1)	41 (24.3)	0.096	− 0.002 (0.960)
HDL-C < 1.2 mmol/L	120 (71.0)	110 (65.1)	114 (64.5)	105 (62.1)	0.060	0.058 (0.093)
Triglyceride > 1.5 mmol/L	29 (17.2)	22 (13.0)	22 (13.0)	26 (15.4)	0.102	0.021 (0.561)
LDL-C > 3.0 mmol/L	74 (43.8)	80 (47.3)	71 (42.0)	65 (38.5)	0.125	0.061 (0.075)
Non HDL-C > 3.37 mmol/L	66 (39.1)	74 (43.8)	63 (37.3)	63 (37.3)	0.006*	0.033 (0.341)

Legend: *SBP* systolic blood pressure, *DBP* diastolic blood pressure, *WC* waist circumference, *WHR* waist to hip ratio, *WHtR* waist to height ratio, *HDL-C* high-density lipoprotein cholesterol, *LDL-C* low-density lipoprotein cholesterol, *GGT* gamma glutamyl transferase, *hs-CRP* highly sensitive c-reactive protein.

Linear regression carried out in age and sex adjusted model showed a significant association between Log_10_ LTL and HDL-C (beta = 0.028; *p* = 0.041) (Table 4) while increasing quartiles of LTL was not associated with HDL-C in linear regression models with similar levels of adjustment. Neither increasing quartiles of LTL nor Log_10_ LTL was associated with other cardio-metabolic parameters in age and sex adjusted linear regression models. In logistic regressions, there were no associations between quartiles of LTL and diabetes, hypertension, any dyslipidaemia and obesity variables. However, when LTL was Log transformed, there was a significant association between Log_10_ LTL and prevalent dyslipidaemia with High LDL-C > 3.0 mmol/L (OR = 0.41, *p* = 0.045) (Table 5).

**Table 4:** Linear regression models (coefficients and standard errors) for the associations of Log_10_ telomere length with cardio-metabolic variables.

Variable	Age (years)	Gender (Sex = reference)	BMI (kg/m^2^)	Log_10_TL	R-squared univariate	R-squared with confounders
SBP	0.663 (0.059)***	− 11.29 (1.935)***	0.337 (0.105)**	5.502 (3.455)	0.004	0.207
DBP	0.23 (0.037)***	− 3.751 (1.201)**	0.358 (0.065)***	2.34 (2.144)	0.002	0.109
TC	0.024 (0.003)***	0.009 (0.102)	0.009 (0.006)	− 0.032 (0.182)	0.000	0.089
TG	0.014 (0.002)***	− 0.298 (0.074)***	0.016 (0.004)***	0.001 (0.132)	0.000	0.095
HDL-C	0.005 (0.001)***	− 0.012 (0.039)	− 0.011 (0.002)***	0.028 (0.041) *	0.002	0.082
LDL-C	0.018 (0.003)***	0.102 (0.08)	0.015 (0.005)**	− 0.13 (0.152)	0.001	0.107
Non HDL-C	0.012 (0.003)***	0.062 (0.09)	0.018 (0.005)***	− 0.13 (0.16)	0.001	0.116

Legend: *SBP* systolic blood pressure, *DBP* diastolic blood pressure, *TC* total cholesterol, *TG* triglyceride, *HDL-C* high density lipoprotein cholesterol, *LDL-C* low density lipoprotein cholesterol, *BMI* body mass index, *TL* telomere length, *** = *p* < 0.001, * = *p* < 0.05.

**Table 5:** Logistic regression models (odds ratios and 95% confidence intervals) for the associations of Log_10_ telomere length with cardio-metabolic conditions.

Variable	Age (years)	Gender (female = reference	Body mass index (kg/m^2^)	Log_10_TL	c-statistics
Obesity (BMI)	1.02 (1.01–1.04)**	0.07 (0.04–0.11)***	–	1.05 (0.47–2.33)	0.54
Hypertension	0.97 (0.95–0.98)***	0.67 (0.42–1.06)	0.97 (0.94–0.99)**	0.63 (0.28–1.43)	0.52
Diabetes	0.95 (0.93–0.97)***	0.72 (0.39–1.36)	0.95 (0.92–0.98)**	0.57 (0.20–1.67)	0.50
High TC > 5.0 mmol/L	0.96 (0.95–0.97)***	1.05 (0.66–1.66)	0.99 (0.97–1.02)	1.34 (0.59–3.02)	0.46
High TG > 1.5 mmol/L	0.96 (0.94–0.97)***	0.33 (0.19–0.58)***	0.96 (0.94–0.99)*	1.09 (0.42–2.88)	0.58
Low HDL-C < 1.2 mmol/L	1.02 (1.01–1.03)**	0.99 (0.66–1.48)	0.95 (0.93–0.97)***	1.16 (0.56–2.40)	0.60
High LDL-C > 3.0 mmol/L	1.04 (1.02–1.05)***	1.22 (0.82–1.83)	1.03 (1.00–1.05)*	0.41 (0.20–0.84)*	0.62
High Non-HDL-C > 3.37 mmol/L	0.96 (0.95–0.98)***	1.10 (0.73–1.65)	0.97 (0.95–0.99)*	1.65 (0.80–3.41)	0.56

Legend: *BMI* body mass index, *SBP* systolic blood pressure, *DBP* diastolic blood pressure, *TC* total cholesterol, *TG* triglyceride, *HDL-C* high density lipoprotein cholesterol, *LDL-C* low density lipoprotein cholesterol, *TL* telomere length, *** = *p* < 0.001, * = *p* < 0.05.

## Discussion

This study examined the associations of LTL with cardiometabolic variables of adiposity, hypertension, type 2 diabetes and dyslipidaemia in a black urban South African population. Linear regression carried out in age and sex adjusted model showed a significant association between Log_10_LTL and HDL-C as continuous variable. In logistic regression model, Log_10_LTL was associated with prevalent dyslipidaemia characterized by high LDL-C. Correlation analysis showed that LTL was associated with Total Cholesterol and non-HDL-C in women and urea in men. However, neither quartiles of LTL nor Log transformed values were associated with hypertension, obesity and type 2 diabetes, which was surprising and warrants further exploration in this population.

The association between LTL and some lipid parameters in the study suggests a possible but weak relationship between shortened TL and dyslipidaemia. Similar results were obtained in the United States^13^ and in Iran^14^. Dyslipidaemia, characterised by altered serum lipid levels is associated with several disease conditions including coronary heart disease, hypertension, diabetes, obesity and oxidative stress which is related with LTL shortening. Although most published literature reported a positive association between short telomere length and a high prevalence of diabetes, obesity, hypertension and other cardiovascular diseases, in this study there were no associations between LTL with diabetes, hypertension or obesity. A meta-analysis of multiple studies found a significant negative association between LTL and diabetes^15^ while a systematic review reported a weak to moderate association between obesity and telomere length^16^. Short TL was also positively correlated with high SBP and DBP^11^, high fasting glycaemia^17^, altered lipid profile markers^6^ and cardiovascular diseases^10,18–20^. The proposed pathway of the association between shortened TL and CMDs reported is bi-directional with cardio-metabolic diseases causing shortening of telomere length and short telomere length increasing the risk of cardio-metabolic diseases. However, these studies have all been carried out in European, Asian and American population, with no studies from Africa, suggesting that the lack of association in our study could be as a result of population differences. Moreover, the sample size of these cross-sectional studies and systematic reviews/meta-analysis was large enough (minimum > 5000) compared to 676 in our study. Therefore, their studies had more power to detect differences compared to our study.

It is possible that LTL could also be determined by the origin and evolution of individuals. Hansen reported shorter TL in Europeans and African Americans originating from Western Africa compared to those living in Africa originating from Tanzania (Eastern Africa)^21^. These results are consistent with other studies reporting longer telomere length in Black Africans compared to white Europeans and Americans in both children and adult^22–27^. However, TL was observed to be longer in white compared to black teachers in South Africa^28^. In this South African population, the risk of cardiovascular disease was higher in black teachers that white teachers^28^. These results shows that genetic differences between ethnic groups and environmental factors ‘contribute’ to overall telomere length.

Unlike this study findings showing no association between TL and age, several studies have reported a significant negative correlation between TL and chronological age. TL shortens with age and age associated disorders. A systematic review showed an average decrease of 21.91 base pair TL/year across cross-sectional studies and 32.2–45.5 base pair TL/year in 5 longitudinal studies^29^. Another important factor that affects TL is sex with several studies showing TL to be longer in women than men^9,30–33^. However, in the present study there was no association between LTL and gender. Even though the present study was carried out in an African population which is different from studies reporting an association between TL and age/gender (Asia, Europe and USA) and could probably explain the difference in the results, further research is needed to explore these associations in Africans. Moreover, the black South African population in which the study was carried out is genetically diverse with some having gene flow from Europe, East Africa and South Asia^34^. This genetic diversity in the study population could be responsible for the difference in the results obtained.

The cross-sectional design of the study prevents conclusions on a causal relationship between LTL and the CMD risk factors investigated. The small sample size of the study and the low sample realisation in men (34%) characteristic of epidemiological studies in this country and probably due to their reluctance to participate, particularly for the drawing of blood samples is another limitation. In this study, multiple hypothesis testing (MHT) was not performed. As such, the results would not withstand a stringent MHT based correction which constitute a possible limitation.

In a black urban South African population, LTL was weakly associated with HDL-C and LDL-C, but not with diabetes, hypertension or obesity. Although the association of LTL with cardiometabolic risk factors have been reported in many populations, there is a paucity of data in African populations. Further research, particularly in longitudinal studies, is required to clearly elucidate the relationship between LTL and CMDs in Africans.

## Materials and methods

### Study site and population

Participants consisted of > 21 years old black men and women residing in Cape Town. This cross-sectional study titled Cardiovascular Risk in Black South Africans (CRIBSA) was conducted in 2008–2009 with data collected by a 3-stage cluster sampling as previously described^35^. This sampling technique was used with quotas, which were pre-specified by age and sex categories to ensure a representative sample, Recruitment took place during office hours and those excluded were the following: pregnant and lactating women, individuals who were bedridden, unable to give consent, on tuberculosis treatment, on antiretroviral therapy, cancer patients having received treatment within the last year and individuals residing in Cape Town for less than three months.

Ethics approval was obtained from the South African Medical Research Council’s Human Research Ethics Committee (EC026-9/2016) and the University of Cape Town’s Research and Ethics Committee (224/2006), and informed consent was obtained from all participants. The research was performed in accordance with the declaration of Helsinki and all methods carried out with relevant guidelines and regulations.

### Data collection

Data, which were collected by trained fieldworkers, included administered questionnaires, clinical examinations and biochemical analyses. Clinical examinations comprised anthropometry (height, weight, and waist and hip circumferences) and blood pressure (BP) measured using standard techniques^36^. A calibrated scale was used to measure weight to the nearest 0.5 kg with each participant barefoot and in light clothing. A stadiometer was used to measure height to the nearest 0.1 cm. A flexible tape measured waist and hip circumferences to the nearest 0.1 cm. For waist circumference (WC), the tape was placed approximately 2 cm above the umbilicus while hip circumference (HC) was measured at the maximum posterior protuberance of the buttocks with the participant standing upright with feet together. Three BP measurements were taken at intervals of 2 min, using an Omron BP monitor after the participant had been rested for at-least 5 min. The average of the second and third BP measurements was used for analysis.

After an overnight fast of approximately 10 h, blood samples were collected by venepuncture into EDTA and dry tubes and a portion processed for biochemical analysis. Plasma glucose (hexokinase) was measured using a colorimetric method according to the manufacturer’s protocol. Total cholesterol (TC), high-density lipoprotein cholesterol (HDL-C) and triglycerides were measured in serum using standard enzymatic techniques^37–39^. Low-density lipoprotein cholesterol (LDL-C) was calculated using the Friedewald formula^40^, while non-HDL-C was calculated using the formula: TC–HDL-C. An oral glucose tolerance test (OGTT) was administered with blood samples collected 2 h after a glucose load^41^. All colorimetric measurements were conducted using a Beckman Coulter AU 500 spectrophotometer. Serum creatinine (CAYMAN CHEMICAL), gamma glutamyl transferase (Abcam) and highly sensitive c-reactive protein (hs-CRP) (BIOMATIK ELISA) measurements were conducted on stored serum samples according to the manufacturer’s protocol. TL assay was conducted from DNA samples extracted from whole blood stored at −80 °C in EDTA tubes using the salt extraction technique. Briefly, 5 mL blood samples in EDTA tubes were defrosted to room temperature and poured into a 50 mL centrifuge tube. Thirty mL lysis buffer (see supplementary material) was added, and red blood cells were lysed by incubation on ice and vortexing. After lysis of red blood cells, the pellets were washed thrice with phosphate buffered saline (see supplementary material) which was later discarded. The pellets were then incubated with nuclear lysis buffer (see supplementary material) overnight at 60 °C. The next day, the supernatant was collected, and the proteins precipitated using 1 mL saturated sodium chloride (6 M) solution. The supernatant containing the DNA was collected into new 15 mL centrifuge tubes and absolute ethanol added to precipitate the DNA by inversion. Precipitated DNA was removed and washed with 70% ethanol. After washing, the precipitate was dissolved in Tris Ethylene Diamine Tetra-Acetate buffer (see supplementary material) and the concentration and quality of the DNA measured using a Nano drop. All samples with absorbance 260 nm/280 nm ratio from 1.7 to 2 were diluted to 5 mg/mL using polymerase chain reaction (PCR) grade water and TL measured by quantitative real time PCR using the method described by O’Callaghan and Fenech^42^.

Serial dilutions of the telomere standard and the single copy gene (36B4) standard were made as described by O’Callaghan and Fenech^42^. A master mix solution containing Power SYBR I (AmpliTaq Gold DNA polymerase, dNTPs, SYBR I Green Dye, optimised buffers and passive reference dye (ROX) (10μL, 1×)), forward primer (1μL, 0.1 μM), reverse primer (1μL, 0.1 μM) and ddH_2_O (4uL) was prepared, mixed well and briefly centrifuged. Using a multichannel pipetted, 16 μL master mix solution were pipetted into each well of a 96 well plate. Into the corresponding wells were added 4 μL each of DNA sample, standards, positive and non-template control (distilled water) in duplicates. The plate was sealed with an optical clear film, centrifuged briefly and run in a QuantStudio 7 Flex Real Time PCR Thermocycler using the following PCR conditions; 10 min at 95 °C, followed by 40 cycles of 95 °C for 15 s 60 °C for 1 min, followed by a dissociation (or melt) curve. At the end of the run, the plate was removed and discarded. Each sample was amplified twice, using telomere forward and reverse primers and the single copy gene forward and reverse primers. After amplification was completed the AB software produced a value for each reaction that is equivalent to kb/reaction based on the telomere standard curve values. The kb/reaction for telomere and genome copies/reaction for diploid genome copy values were exported and used to calculate the LTL in kilobase (kb) as follows; LTL = $\frac{{{\text{telomere}}\;{\text{kilobase}}\;{\text{per}}\;{\text{reaction}}\;{\text{value}}}}{{{\text{diploid}}\;{\text{genome}}\;{\text{copy}}\;{\text{number}}}}$.

### Definitions

Body mass index (BMI), calculated as weight (kg)/height (m) squared, classified participants into three categories of generalised adiposity: normal weight (18 ≤ BMI < 25 kg/m^2^), overweight (25 ≤ BMI < 30 kg/m^2^) and obese (BMI ≥ 30 kg/m^2^)^43^. Central obesity was determined using the following criteria: WC > 94 cm in men and > 80 cm in women, waist-to-hip ratio (WHR) ≥ 0.9 in men and ≥ 0.85 in women^44^ or waist-to-height ratio (WHtR) > 0.5^45^. Hypertension was defined as systolic BP (SBP) ≥ 140 mmHg or diastolic BP (DBP) ≥ 90 mmHg or known hypertension on treatment^46^. Dyslipidaemia was defined as TC > 5 mmol/l, triglycerides > 1.5 mmol/L, HDL-C < 1.2 mmol/L, LDL-C > 3.0 mmol/L and non-HDL-C > 3.37 mmol/L or taking anti-lipid agents^47^. Diabetes was defined as fasting plasma glucose ≥ 7.0 mmol/L and/ or 2-h post glucose load ≥ 11.1 mmol/L, previously diagnosed or taking antidiabetic medications^41^.

### Statistical analysis

Data analysis was carried out using SPSS Version 21 software. Continuous variables are presented as medians (25th to 75th percentiles) and categorical variables as counts (percentages). Mann Whitney U test was used to compare baseline characteristics by sex. TL was categorised into quartiles and the linear trend in CMD profile (continuous variables) across the different quartiles of TL was computed using the median test. Similarly, chi square test was computed and the linear-by-linear association used to compare the trend in proportions of disease conditions (categorical variable) across the quartiles of TL. Spearman correlation was used to assess the association between quartile of TL and cardio-metabolic parameters. The interactions between TL categories and cardio-metabolic risk profile were tested using linear and logistic regressions, by incorporating in the same model the main effects of the variables of interest as well as their interaction term with TL. In linear and logistic regression analyses, TL was log transformed. A *p* value < 0.05 was considered statistically significant.

## Supplementary Information

Supplementary Information.

## Data Availability

The datasets generated during and/or analysed during the current study are available from the corresponding author on reasonable request.
