# Supplementary material for: Cardiometabolic profile and leukocyte telomere length in a Black South African population
Source: Sci Rep. 2022 Feb 28;12:3323. doi: 10.1038/s41598-022-07328-8 (PMC8885820; doi:10.1038/s41598-022-07328-8)
Supplement: Supplementary file 1 — Supplementary Information. [file 41598_2022_7328_MOESM1_ESM.docx]

**Buffer preparation for DNA extraction using the salting out method**

**Red Blood Cell Lysis buffer (1L)**

155mL of NH_4_Cl (1M) + 10mL of KHCO_3_ (1M) + 1mL EDTA (100mM) and make up to the volume to 1 L solution while adjusting the pH to 7.4.

**Nuclear Lysis Buffer (400mL)**

4mL of Tris (1M) + 9.2g of NaCl + 8mL of EDTA (0.1M) and make up the volume to 400mL with distilled water adjusting the pH to 8.2

**Phosphate Buffered Saline (PBS)**

0.2g KCl + 8.0g NaCl + 0.2g KH_2_PO_4_ + 1.15g Na_2_HPO_4_ dissolved in double distilled water and made up to 1L solution while adjusting the pH to 7.4.

**Tris EDTA Buffer (1X, 100mL)**

Pour 80mL solution in a clean flask and add 0.158g Tris-Cl + 0.029g EDTA and complete the volume to 100mL while adjusting the pH to 8.0.
